# Supplementary material for: In Vitro Effects of Pesticides on European Foulbrood in Honeybee Larvae
Source: Insects. 2020 Apr 17;11(4):252. doi: 10.3390/insects11040252 (PMC7240397; doi:10.3390/insects11040252)
Supplement: Supplementary file 1 [file insects-11-00252-s001.zip › SupplementaryTables_Figures.pdf]

insects

MDPI

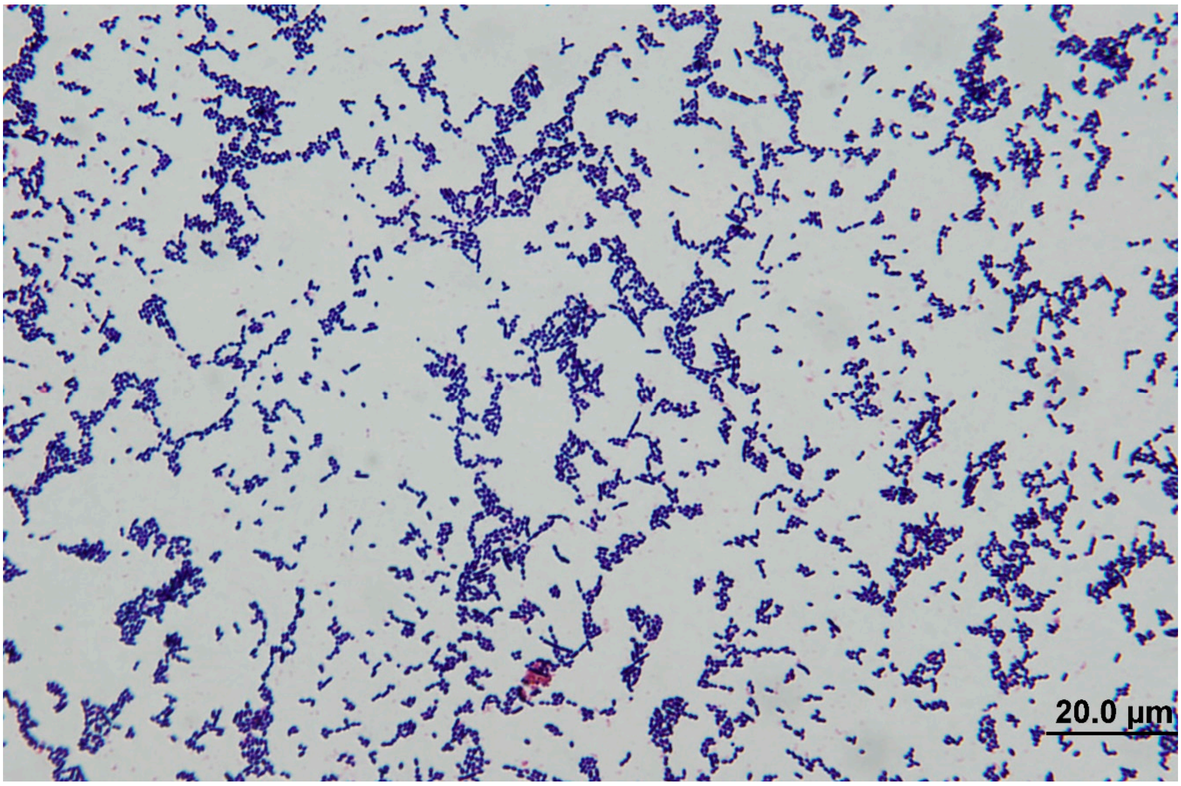

Figure S1. Gram stain of *Melissococcus plutonius* isolate from a honey bee colony pollinating blueberries in the Fraser Valley of British Columbia. The bacteria forms characteristic short chains of gram-positive lanceolate cocci.

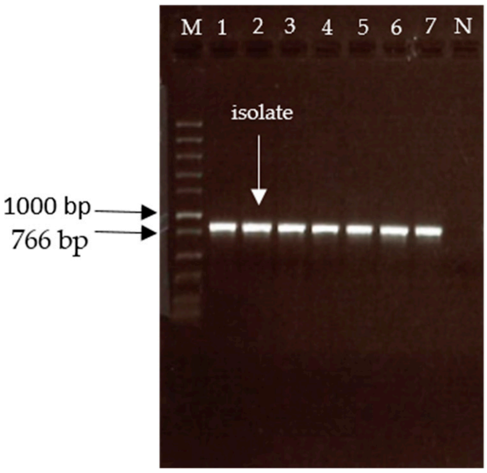

Figure S2. Agarose gel (1%) of PCR product from genomic DNA of *Melissococcus plutonius* isolates. The white arrow indicates an 812 base pair band which confirms the *M. plutonius* identity of the bacterial isolate utilized in the present study. Lanes 1, 3, and 4-7 represent other *M. plutonius* isolates; N, no template control; M, molecular size marker (Fast DNA Ladder, New England BioLabs, Whitby, ON, Canada).

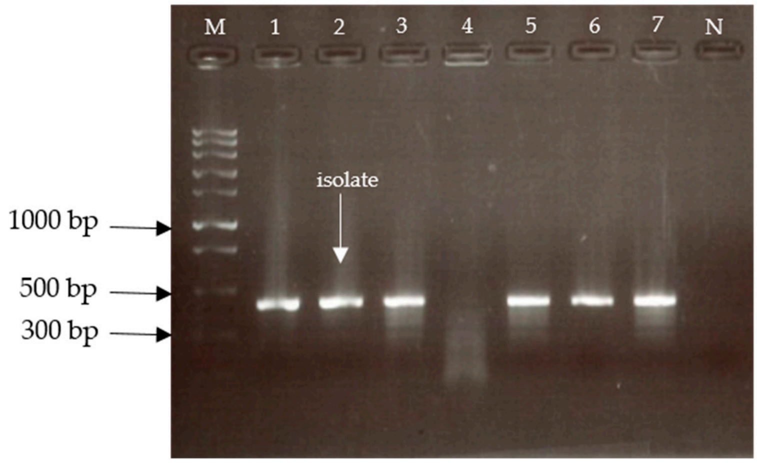

Figure S3. Agarose gel (1%) of duplex PCR product from genomic DNA of *Melissococcus plutonius* isolates. The white arrow in lane 2 indicates a 424 base pair band which identifies the bacterial isolate used in the present study as an atypical strain of *M. plutonius*. Lanes 1, 3, and 5-7 represent other atypical *M. plutonius* isolates; lane 4 is a PCR reaction failure; N, no template control; M, molecular size marker (Fast DNA Ladder, New England BioLabs, Whitby, ON, Canada).

Table S1. Cox proportional hazards regression survival analysis of 46-84 *Apis mellifera* larvae infected with 50 colony forming units (CFU) of *Melissococcus plutonius* and chronically exposed dietary thiamethoxam and/or fungicides from day 0 to day 5 *in vitro*. Mortality was recorded daily for 6 days.

				**95% Confidence Interval**	
**Treatment (ng/µl)**	**Hazard ratio[1]**	**Standard error**	**P**	**lower**	**upper**
THI 1	1.52	0.46	0.17	0.84	2.74
THI 10	1.35	0.38	0.29	0.78	2.33
BOS 29	1.16	0.25	0.48	0.77	1.76
THI 1 BOS 29	0.97	0.19	0.88	0.66	1.44
PYR 14	0.91	0.26	0.75	0.53	1.59
THI 1 PYR 14	0.76	0.21	0.33	0.44	1.32
PROP 14	1.57	0.51	0.17	0.83	2.98
THI 1 PROP 14	1.85	0.58	0.048	1.00	3.42

[1]The hazard ratio indicates the effect of a unit change in pesticide treatment on frequency of death relative to the control group which was infected with 50 CFU *M. plutonius* and received uncontaminated diet from day 0 to day 5. THI, thiamethoxam; BOS, boscalid; PYR, pyrimethanil; PROP, propiconazole.
